# Mental health policy and system preparedness to respond to COVID-19 and other health emergencies: a case study of four African countries

**DOI:** 10.1177/00812463211012177

**Published:** 2021-06

**Authors:** Tholene Sodi, Mahlatse Modipane, Kwaku Oppong Asante, Emmanuel Nii-Boye Quarshie, Stephen Asatsa, Julia Mutambara, Sibusiso Khombo

**Affiliations:** 1 University of Limpopo, South Africa; 2 University of Ghana, Ghana; 3 The Catholic University of Eastern Africa, Kenya; 4 Midlands State University, Zimbabwe

**Keywords:** Africa, COVID-19, health emergencies, health systems, mental health systems

## Abstract

As a result of a long colonial history and subsequent developmental and economic challenges, many African countries have struggled to put in place adequate policies, systems, and associated infrastructures to address the health and social needs of their citizens. With the COVID-19 pandemic threatening human lives and livelihoods, concerns are raised about the preparedness and readiness of health policies and systems in African countries to deal with these kinds of health calamities. More particularly, questions can be asked about the preparedness or even existence of mental health policies and associated systems to help individuals and communities in Africa to deal with the consequences of COVID-19 and other health emergencies. In this article, we analyse the existing mental health policies of four African countries paying attention to the capacity of these legislative provisions to enable psychology professionals to deal with psychosocial problems brought about by COVID-19. We use Walt and Gilson’s Policy Triangle Framework to frame our analysis of the existing mental health policies. In line with this conceptual framework, we review the role played by the different factors in shaping and influencing these mental health policies. We further explore the challenges and opportunities associated with existing legislation and mental health policies. We also reflect on the reports obtained from each of the four countries about the role that psychologists are playing to deal with the associated psychosocial problems. Based on our policy analysis and country reports, we highlight strengths and gaps in these policies and give recommendations on how mental health policies in these countries can be strengthened to respond to COVID-19 and future health emergencies.

Since the outbreak of the coronavirus disease (commonly known as COVID-19) in December 2019, there have been an estimated 29,155,581 people infected, with 926,544 deaths reported, by the time of preparation of this article (World Health Organization [WHO], 2020). In view of its devastating impact, the WHO declared COVID-19 a pandemic on March 11, 2020. Studies have indicated that the pandemic has resulted in many social and economic problems for individuals, families, and communities. At the individual level, it has been noted that COVID-19 has had immense psychological consequences ranging from anxiety among school children due to prolonged closure of schools, guilt and fear among health care workers, and grief complications due to restricted mourning activities (Otanga, 2020; Serafini et al., 2020). As the pandemic worsened, there were concerns raised about the preparedness of African health care systems to cope with the potentially disastrous health and social consequences of COVID-19. The poor health care systems on the continent are usually blamed on the long colonial history and subsequent developmental and economic challenges (Ochen & Nwanko, 2012). As a consequence, many African countries have struggled to put in place adequate policies, systems, and associated infrastructures to address the health and social needs of their citizens. More particularly, questions may be asked about the preparedness, or even existence of mental health care policies and associated systems to help individuals and communities in Africa to deal with the psychological fallout of COVID-19.

The purpose of this article is to examine the preparedness of mental health legislation and policies in four African countries (namely, Ghana, Kenya, South Africa, and Zimbabwe) in responding to COVID-19 and other health emergencies. We start by reviewing the existing mental health policies of these countries, paying attention to the capacity of their legislative provisions to enable psychology professionals to deal with psychosocial problems brought about by COVID-19. In the second part of the article, we use Walt and Gilson’s (1994) Policy Triangle Framework to frame our analysis of the existing mental health policies. In line with this conceptual framework, we critically review the role played by the different factors (that is, actors, context, content, and process) in shaping and influencing these mental health policies. In the third part of the article, we explore the challenges and opportunities associated with existing legislation and mental health policies. In the fourth part, we reflect on the reports from each of the four countries on the role that psychology professionals are playing at local and national levels to deal with the psychosocial problems associated with the pandemic. Based on our policy analysis and country reports, we draw our conclusions by highlighting the strengths and gaps in these policies and make recommendations on how mental health policies in these countries can be strengthened to respond to COVID-19 and future health emergencies.

## Overview of existing policies and their recognition of the profession of psychology

In all the four countries, there exist legislation and policies that make provision for mental health services and the psychology profession (see Table 1).

**Table 1.** Existing mental health legislation and policies in Ghana, Kenya, South Africa, and Zimbabwe.

Country	Legislation/policy	Goal of legislation/policy
Ghana	Ghana’s Mental Health Act (Act 846, Mental Health Act, 2012)	• The act ensure that persons with mental disorders receive right and quality treatment • Makes provision for the funding of mental health services in Ghana
Kenya	Counsellors and Psychologists Act 2014 (Republic of Kenya, 2014)	• Provides for registration and licencing of counsellors and Psychologists • Establishes a counsellors and Psychologists Board under the Ministry of Health to regulate Counsellors and Psychologists
	Kenya Mental Health Policy 2015–2030 (Ministry of Health, 2015)	• Provides guiding principles, human resource management, community-level engagement, and public-private partnerships in the provision of mental health services
	Public Service Guidance and Counselling Policy (Ministry of Health, 2017)	• Provides the guidelines for provision of mental health services to the public sector
	Mental Health Act, 1989 (Republic of Kenya, 1989)	• Provides for care and management of persons suffering from mental illnesses
South Africa	Mental Health Care Act (MHCA) Number 17 of 2002 (Source: Department of Health, 2002)	• The act provides for the care, treatment, and rehabilitation of persons who are mentally ill • Makes provision for the integration of mental health into primary health care
	Health Professions Act, Number 56 of 1974 (Source: Department of Health, 1974)	• Establish the Health Professions Council of South Africa which is responsible for regulating health professions falling within its ambit.
Zimbabwe	Mental Health Act 1996 [Chapter 15:12] (Source: Ministry of Health and Child Welfare)	• The act provides for the care of mentally ill patients through institutionalisation • It seeks to safeguard the rights of mental health patients
	Zimbabwe `s Mental Health Policy 2007 (Source: Ministry of Health and Child Welfare)	• Aims to provide a comprehensive, well-coordinated quality mental health service to all Zimbabweans. • It also seeks to integrate mental health services into the general medical health system with the aim of improving the mental health of the nation
	Zimbabwe National Strategic Plan for Mental Health Services 2014-2018 (Source: Ministry of Health and Child Care)	• The policy gives a framework within which mental health programmes, projects, and activities can be designed and implemented, monitored, and evaluated

Below, we give a brief overview of the legislative and policy provisions for each country:

### Ghana

Prior to the passage of the Mental Health Act (Act 846, 2012) in Ghana, only a few clinical psychologists were engaged for the provision of mental health care in regional and referral hospitals. However, since 2012, there has been a considerable increase in the placement of clinical psychologists in regional and district hospitals across the country. In particular, the Act identifies various mental health workers as key role-players in the provision of mental health care in the country: psychiatrists, clinical psychologists, mental health nurses, social workers, and other appropriately trained or qualified persons with specific skills relevant to mental health care. Although the Act provides for the use of a certificate of urgency in mental health care (Anokye et al., 2018), it makes no provision to enable psychologists and other mental health care professionals to deal with psychosocial challenges brought about by once-off emergencies (e.g., fires, floods, and accidents) or prolonged public (mental) health crises/pandemics like COVID-19. A recent experience with a combined flood and fire emergency suggests that mental health professionals only volunteer *ad hoc* emergency care to help ameliorate trauma-induced distress (Quarshie et al., 2018).

### Kenya

The Counsellors and Psychologists Act 2014 (Republic of Kenya, 2014) lays down the framework for training, registration, and regulation of counsellors and psychologists in Kenya. The Act provides for the establishment of a psychologists’ board housed in the Ministry of Health whose mandate it is to regulate the counselling and psychology profession. However, since its enactment, the Act has not been implemented. A board is not in existence, and this leaves counsellors and psychologists to operate unchecked. With the absence of a central command centre for psychologists, a coordinated response to humanitarian psychosocial problems has been very poor. This gap has occasionally been filled by humanitarian and non-governmental organisations who usually step in to work together with psychologists’ associations in the country to reach the masses. The Mental Health Act 1989 (Republic of Kenya, 1989) has also attempted to regulate the work of psychiatrists and psychologists but some of its sections remain unimplemented. For instance, the Act, proposes the establishment of district mental health councils as an attempt to bring mental health services to the people. However, the taskforce on mental health (Ministry of Health, 2020a) observes that this has never been actualized with only 26 out of the 47 counties having psychiatric units. In responding to COVID-19, the existing laws and policies have not been so helpful, prompting the Ministry of Health to come up with Standard Operating Procedures for Counsellors and Psychologists providing Mental Health and Psychosocial Support for COVID-19 responses in Kenya (Ministry of Health, 2020b). This has managed to mobilise a few mental health professionals to offer help in isolation centres and hospitals. However, the large number of psychologists in the country who could be tapped to provide community intervention remain uncoordinated with mental health–related issues gradually increasing during the COVID-19 pandemic.

### South Africa

Both the Health Professions Act (Department of Health, 1974) and the Mental Health Care Act Number 17 of 2002 (Department of Health, 2002) make provision for different categories of health workers to provide a wide range of health services to individuals and communities. The Health Professions Act, in particular, provides for the establishment of the Professional Board for Psychology which has the powers, among others, to have oversight control and to exercise authority in respect of all matters affecting the education, training, and practice of psychology professionals (Health Professions Council of South Africa, 2021). The professions currently registered under the auspices of the Professional Board for Psychology are psychologists, registered counsellors, psychometrists, and psychotechnicians. With regard to psychologists, the Health Professions Act recognises the following categories of registration: clinical, counselling, educational, industrial, and research psychology (Department of Health, 1974). The category of neuropsychology has recently been added to the register of psychologists (Health Professions Council of South Africa, 2021).

### Zimbabwe

The categories of mental health personnel in Zimbabwe include clinical psychologists and community psychologists. Counselling psychologists are a newer group, and although none are registered yet, more than 50 are currently doing internships. Many social workers are being trained, and during the pandemic, some were recruited to work at quarantine centres. The mental health policies and strategies give room for psychologists to operate. The mental health policy notes that psychotherapy, counselling, behaviour therapy, and psychosocial treatment and rehabilitative therapy should be available in mental health institutions. However, the aim is more curative (Ministry of Health and Child Welfare, 2007). With crisis events like the COVID-19 pandemic and its nationwide coverage, there is a need to focus more on prevention and promotion with a community lens – a role which can be filled by community psychologists. Although the policy documents facilitate the provision of mental health services, the major drawback has been the issue of human and material resource constraints (Kidia et al., 2017). Workforce challenges have intensified in Zimbabwe because of the adverse economic and political climate. Human and material resource constraints hinder the development of community-based interventions that build capacity and promote task-shifting among non-specialist providers (Liang et al., 2016).

## Using Walt and Gilson’s (1994) policy triangle framework to analyse existing mental health policies

**Figure 1.**
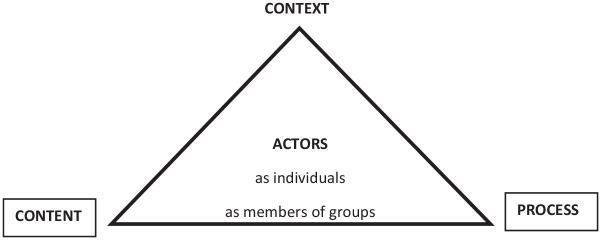
A model for health policy analysis (Source: Walt & Gilson, 1994).

There exist a number of frameworks and theories for analysing public policy and related processes (Gilson & Raphaely, 2007). These theoretical tools help to frame policy analysis by identifying critical elements and the relationships among these elements. In this article, we opted to use Walt and Gilson’s (1994) Policy Triangle Framework, which was specifically developed for health policy analysis (Walt & Gilson, 1994). The framework identifies context, actors, content, and processes as the four key elements that interact to shape policy-making (see Figure 1).

According to Gilson and Raphaely (2007), Walt and Gilson’s framework has influenced policy research in many countries and has been used to analyse health issues such as mental health and reproductive health. Through this framework, we identify and critically discuss the four key elements (i.e., context, actors, content, and processes) characterising the mental health legislation and policies in the four African countries.

### Context

The four countries whose mental health legislation and policies are the focus of our analysis have all emerged from many years of colonialism, with Ghana attaining its independence in 1957 (Heldring & Robinson, 2013) while Kenya’s independence was achieved in 1963 (Kimani, 1963). The two countries in the southern tip of Africa attained their independence much later, with Zimbabwe gaining independence in 1980 (Law, 2016), while South Africa’s democratic dispensation was in 1994. What is common in these four countries is that prior to independence, mental health care was a low priority, with the majority of the population having little or no access to mental health services. For instance, in Zimbabwe, there was very little training for mental health because only one facility had been committed to mental health service, which may suggest that a number of those living with psychiatric conditions received no care (Ministry of Health and Child Care [MOHCC], 1984). Since independence, each of the four counties has introduced legislation and policies specifically to deal with mental health problems. In Ghana for instance, these legislative and policy initiatives were mainly influenced by the rising incidents of mental health problems at the population level (e.g., about 21% of the general adult population reported moderate or severe psychological distress [Canavan et al., 2013]). While the legislative and policy initiatives have provided a broader framework for the delivery of mental health services, the lingering effects of colonialism (in Ghana, Kenya, and Zimbabwe) and apartheid policies (in South Africa) have continued to negatively impact the development and delivery of meaningful services in these countries. For example, in South Africa, provision of health care services, more especially mental health care, has continued to be skewed in favour of the white minority (Gordon et al., 2020; Maphumulo & Bhengu, 2019). The implication here is that even though mental health care legislation is in place, the services envisaged in these policy documents are not likely to be accessible to the majority of the population.

### The actors

Looking at the four countries, it is evident that the formulation of mental health care policies has been a product of various actors ranging from the political class, mental health professionals, and the (lay) community. Invariably, the politicians, though not mental health experts, tend to drive the process of legislating important laws to regulate mental health through acts of parliament. The government, usually through a health department, tends to play a facilitative role in the implementation of these mental health policies and frameworks, once legislated. The different categories of mental health professionals in both public and private sectors, the non-governmental organisations providing mental health care and related services, and the users of mental health services all appear to play a role in the development of mental health legislation and policies. For instance, in Ghana, the actors who played a role in the mental health policy development were the Ministry of Health, with support from the WHO, and a wide range of key stakeholders, including mental health professionals, general health workers, law experts, traditional and faith-based healers, and teachers (Osei et al., 2011). In all these countries, it appears that professional associations such as the Ghana Psychological Association (GPA), the Zimbabwean Psychological Association, and the Psychological Society of South Africa are also playing a critical role in mental health policy development and implementation. In Kenya, the potential role of the associations appears hindered by the fact that there are a few competing associations, with the result that there is no single nationally representative organisation for psychologists.

### Content

The Mental Health Act, 2012 (Act 846) of Ghana (Mental Health Act, 2012), the Mental Health Amendment Bill (Republic of Kenya, 2018); Counsellors and Psychologist Act of 2014 in Kenya (Republic of Kenya, 2014, 2018), the Mental Health Act of 2002 and Health Professions Act of 1974 (Department of Health, 1974, 2002) in South Africa, and the Zimbabwe National Mental Health Policy (MOHCC, 2019), are pieces of legislation that are aimed at (1) providing a legal framework to guide the safe management of patients and (2) establishing some kind of authority to oversee and regulate mental health services in the respective country (Doku et al., 2012; Osei et al., 2011; Walker & Osei, 2017). As we have already pointed out, these existing mental health policies tend to make provision for psychologists and other categories of mental health professionals to play significant roles in mental health care provision, including the delivery of professional services that are required during health emergencies. In South Africa, for example, the scope of the profession of psychology allows for psychology professionals to, among others, use:

. . . any psychological method or practice aimed at aiding persons or groups of persons in the adjustment of personality, emotional or behavioural problems or at the promotion of positive personality change, growth and development, and the identification and evaluation of personality dynamics and personality functioning according to scientific psychological methods. (Health Professions Council of South Africa, 2008, pp. 5–9)

The scope of psychology professionals makes provision for a practitioner to provide short-term mental services such as psychological screening, primary mental status screening and psychological interventions that are aimed at enhancing personal functioning. These are the kinds of services that are commonly needed during times of health emergencies. Although the legislation and policies in the four countries provide for the existence of psychology professionals, it appears that the main challenge relates to training and retaining a sufficient number of these mental health practitioners to deliver the service in both public and private sectors (Mangezi & Chibanda, 2010).

### Process

In all four countries, the process of policy formulation is rigorous with various stakeholders being involved. In Zimbabwe, for example, the process of formulation of the current Zimbabwe National Mental Health Strategic Plan (ZNMHSP) 2019–2023 strategy involved a review of the strategic plan 2014–2018. Achievements and challenges of the last 4 years (covered by the previous Strategic plan) were discussed to aid the development of a new strategic plan, and an analysis of the strengths, weaknesses, opportunities and threats (SWOT analysis) was done (MOHCC, 2019). Needs analyses were done through community engagement and these were then incorporated in the policy. However, most of the community’s ideas may not be taken into consideration, as they are usually seen by the policy implementers as having ‘overly optimistic expectations’ (Kagstrom & Dovlen, 2019), and because of that, health services remain ‘alien’ to the general public.

## Challenges and opportunities associated with existing legislation and mental health policies

The legislation and mental health policies in the four African countries provide both challenges and opportunities, some of which we will briefly outline below.

### Challenges in the effective implementation of legislation and mental health policies

The existing legislation and mental health policies that each of the four African countries have put in place appear adequate in so far as they enable psychology professionals to provide professional services to individuals, families, and communities under ordinary circumstances and during times of crisis. In terms of Walt and Gilson’s (1994) Policy Triangle Framework, it appears that the shortage of psychology professionals (actors) and contextual and process factors are the key hindrances in the provision of psychological services during health emergencies. For example, a study by Hlongwa and Sibiya (2019) found that one of the challenges in mental health care delivery in South Africa is the lack of human, financial, and infrastructural resources. This means that even though policies are in place, there is, for instance, an insufficient number of psychologists to provide the necessary services. The shortage of psychology professionals and other mental health professionals has also been highlighted by the WHO – the number of psychologists in African countries is very low (WHO, 2014). For example, in Ghana the ratio of psychologists to the population was reported to be 0.07 per 100,000 people (WHO, 2017). This is marginally higher than in Zimbabwe where the ratio was reported to be 0.06 per 100,000 people during the same period.

### Policies providing an enabling environment

Generally, the different pieces of legislation in the four countries, including the associated mental health policies are fairly comprehensive and reasonably fit-for-purpose. For example, Ghana’s current mental health legislation (i.e., Act 846 of 2012) is more comprehensive as it provides for the protection of the rights of patients and mental health service users, it ensures ethical oversight of treatment, and makes specific arrangement for funding of mental health care in the country (Doku et al., 2012; Osei et al., 2011; Walker & Osei, 2017). In Kenya, some of the fully operationalised mental health policies such as the Counsellors and Psychologists Act, 2014, have proved to be enablers of mental health service provision in various sectors as cited by the mental health task force (Ministry of Health, 2020a). In Zimbabwe, the current strategic plan has led to the convening of the National Mental Health Taskforce and the Provincial Mental Health Taskforces (MOHCC, 2019). These teams are made up of private and public role-players. During the COVID-19 pandemic, for example, the Midlands Province Mental Health team consisting of mental health practitioners in the province developed Information, Education, and Communication (IEC) material for those in quarantine centres and health workers. This was after it was observed that mental health issues were not being addressed by the Provincial COVID-19 Taskforce. The mental health strategy further aims to strengthen activities that are relevant to the context and has proposed the adoption of the Friendship Bench (FB) – a countrywide initiative that provides mental health through task-shifting using the services of elders and lay people (MOHCC, 2019). In South Africa, the National Department of Health developed the mental health guidelines during the COVID-19 pandemic with the aim of providing information to promote and protect the mental well-being of the citizenry; and to raise awareness about mental disorders and mental health problems that may arise due to the COVID-19 (National Department of Health, 2020). The guidelines also direct health care facility managers, health care providers, and informal caregivers on actions they need to take to identify and manage mental health problems that may arise out of COVID-19.

## The role of psychologists during the COVID-19 outbreak

In this section, we present our observations regarding the role of psychology professionals in the four countries during the COVID-19 pandemic. We do this by presenting a synopsis of the kinds of services that these mental health professionals were called upon to provide to help contain the health emergency.

### Ghana

During the earlier weeks of the virus in the country, under the guidance of the GPA, three professional psychologists were invited to speak and educate the Ghanaian public on practical steps to mitigate the distress of lockdown and physical distancing; how the general population should interact with persons recovering from COVID-19; and how various sectors of society should handle COVID-19 matters to prevent stigmatisation. Furthermore, an experienced clinical psychologist was appointed as an Advisor to the National COVID-19 Response Team, to help provide expert advice on possible psychosocial issues that need to influence government intervention efforts in addressing the psychosocial and economic needs of the population. The GPA, through the Ghana Health Service, with support from the Ministry of Health, established the GPA COVID-19 Response Team to provide psychosocial support to deportees/returnees, international students, and travellers who must undergo mandatory quarantine. Also, clinical psychologists have been included in the medical teams at the various COVID-19 treatment facilities and centres. The clinical psychologists provide emotional care and general psychological support to patients undergoing treatment of the virus.

### Kenya

The Ministry of Health directed counsellors to use Psychological First Aid as the standard intervention model, as noted in the Standard Operating Procedures for Counsellors and Psychologists providing Mental Health and Psychosocial Support for the COVID-19 response in Kenya (Ministry of Health, 2020b). With the emphasis on the isolation centres, community distress was left to the counsellors and psychologists in private practice. Some counsellors developed online therapy platforms for the provision of therapy and seminars, while others have been hosted on the mainstream media to educate the public. Continuous education thrived as counsellors and psychologists grouped into virtual teams and conducted daily trainings on specific areas related to COVID-19. However, the uptake of counselling services remained low due to the cost implication.

### South Africa

Since the outbreak of COVID-19, psychologists in South Africa have individually and collectively availed their services in support of the government’s efforts to manage the pandemic. For instance, the Psychological Society of South Africa (PsySSA), a national membership organisation of psychology professionals and others involved in the discipline, has teamed up with other organisations to form what is known as the HealthCare Workers Care Network (HWCN). This group of professionals is made up of psychologists, psychiatrists, anaesthesiologists, medical doctors, and other health professionals with the aim of supporting health care workers during the COVID-19 pandemic and beyond (Psychological Society of South Africa, 2020).

At the community level, psychologists and registered counsellors are also involved in providing psychosocial support to vulnerable individuals and groups such as homeless people, the elderly, children, and those exposed to violence, drugs, and substance abuse. These efforts are coordinated by the national and provincial health departments and nongovernmental organisations. The government and the private sector are also using various platforms, such as radio, television, and the media, to offer mental health education to communities.

To promote wider access to psychological services during the pandemic, the Health Professions Council of South Africa (HPCSA) revised its telehealth policy to allow psychologists to provide psychological services remotely even in instances where no prior practitioner–patient relationship existed (Health Professions Council of South Africa, 2020). It is envisaged that this form of intervention is an avenue that may provide an opportunity for psychologists in the future to reach an even wider segment of the South African society.

### Zimbabwe

The National Mental health taskforce is offering training for mental health cadres to be able to deal with issues on the ground. Provincial Mental Health Teams are coordinating activities with quarantine centres and offering referrals to those who need medication. Due to staff shortages, counselling services are not being adequately provided. Mental health education (e.g., on COVID-19 induced anxiety, depression, and suicide), seminars and discussions are also being offered on various (social) media platforms such as radio, television *WhatsApp, Faceboo*k, *Zoom*, and *Googl*e *Meet*. The response has been overwhelming. Some of these online platforms have also provided information on the psychosocial impact and coping strategies from survivors of COVID-19.

## Gaps, strengths, and recommendations

Based on our analysis, we identified the following gaps and strengths inherent in the mental health legislation and policies of these four African countries:

### Gaps

Looking at the four countries, it appears that the historical legacies of colonialism and apartheid have continued to have a negative impact on the implementation of mental health legislation and policies. Some of the common problems in all these countries are inaccessibility of mental health services due to lack of prioritisation of mental health services and the failure to implement what is articulated in the policies and Acts (Kagstrom & Dovlen, 2019; Liang et al., 2016), as well as low budgets for mental health services (Kidia et al., 2017; Mangezi & Chibanda, 2010; WHO, 2014, 2017). In South Africa, for example, the current funding formula for mental health services has been found to be significantly inadequate, resulting in a lack of psychological services more especially in schools, and at clinic and community mental health care levels (Docrat et al., 2019). It is these kinds of challenges that make it difficult for mental health services to be accessible to individuals and communities (Monterio, 2015). In Kenya, the biggest problem is failure to implement the available mental health legislations, therefore, no regulation and funding of mental health services are available. This was observed by the mental health taskforce of 2020 (Ministry of Health, 2020a). In Zimbabwe, these kinds of problems are compounded by the current political and economic environment that hampers efforts by psychologists and other mental health professionals towards providing psychosocial support to the general population. The problems highlighted here have the potential to hinder mental health care systems in their effort to deal with health problems, including current and future health emergencies.

### Strengths

Despite the challenges and gaps highlighted above, all four African countries have mental health legislation and policies in place. In all instances, provision is made in these policies for the existence of the psychology profession. In South Africa, for example, the scope of the profession of psychology provides for the different categories of psychology professionals who are empowered to provide their services at individual, family, and community level (Health Professions Council of South Africa, 2008). The relaxation of legislation and policies in some of these countries by allowing for delivery of mental health services remotely through technology has the potential to make mental health services more accessible to more people. For example, in South Africa, the Health Professions Council of South Africa (HPCSA) – a statutory body regulating the profession of psychology – allowed for telepsychology to be offered in response to COVID-19. In a recent notice issued on March 26, 2020, the HPCSA pronounced that:

‘Telehealth is only permissible in circumstances where there is an already established practitioner-patient relationship, except where telepsychology and/or telepsychiatry is involved, in which case telehealth is permissible even without an established practitioner-patient relationship’. (Health Professions Council of South Africa, 2020)

### Recommendations

Based on our review of the legislation and mental health policies of the four African countries, we make the following recommendations:

1. Specific provision should be made in the legislation and mental health policies to offer mental health care services during both once-off and prolonged public health emergencies. This means that the existing policies should be broadened to explicitly outline the nature and type of services that could be provided during health emergencies. In this regard, psychology as a behavioural and human science has a greater role to play. As Pillay and Barnes succinctly put it, psychology ‘. . . is well placed to advise on necessary social policy development especially considering that societal support is essential for governments to effectively manage a pandemic’ (Pillay & Barnes, 2020, p. 152).
2. Consideration should be given to introducing more mental health services that can be delivered remotely through technology. Telepsychology has the potential to make services available to the majority of people in remote areas. In line with recommendations made by De Sousa et al. (2020), it is proposed that health authorities consider the adoption, inclusion, and application of culturally sensitive, ethically sound, and quality-assured tele-mental health care approaches, to not only broaden the coverage and access to mental health care, but to also strengthen response to mental health challenges, including those occasioned by COVID-19 and future public (mental) health emergencies.
3. Given the multifaceted nature of mental health problems, more efforts should be made to encourage various mental health professionals (psychiatrists, psychologists, counsellors, and other community health care professionals) to work collaboratively but less competitively, in the interest of the public.
4. Coordination of mental health response in times of crisis should be done by the respective countries’ departments of health with the active participation of other stakeholders such as health professional associations and nongovernmental organisations.
5. It is recommended that more community-oriented psychological interventions be introduced. These would complement the predominantly individual-oriented psychotherapeutic modalities that have hitherto been the standard interventions. Embracing more community-oriented interventions has the potential to make psychological and other mental health services more accessible to the majority of people. Mobile mental health care centres need to be established. For instance, during the implementation of the AIDS policy in Zimbabwe, there were village and ward cordinators who interracted with community members (National AIDS Council, 2006). A similar approach could be used for mental health services, more epecially during pandemics such as COVID-19.
6. There is a need for the policy implementers to recognise the complex link between mental health and poverty. Thus, the development of mental health programmes should consider the different socioeconomic levels of the population. This is particularly important, as several people across the subregion have lost their sources of income as a result of the COVID-19 pandemic. This has led to depression, anxiety, and other mental health problems.
7. Consideration should also be given to allocating resources for research, so as to help in discovering new and more effective response models to deal with COVID-19 and other future health emergencies.
